# Effect of Hostile Solutions on the Residual Fatigue Life of Kevlar/Epoxy Composites after Impact Loading

**DOI:** 10.3390/molecules26185520

**Published:** 2021-09-11

**Authors:** Paulo N. B. Reis, Marco P. Silva, Paulo Santos, João Parente, Sara Valvez

**Affiliations:** 1 Department of Mechanical Engineering, CEMMPRE, University of Coimbra, 3030-788 Coimbra, Portugal; 2 C-MAST, Department of Electromechanical Engineering, University of Beira Interior, Calçada Fonte do Lameiro, 6201-100 Covilhã, Portugal; marco.silva@ubi.pt (M.P.S.); paulo.sergio.santos@ubi.pt (P.S.); joao.miguel.parente@ubi.pt (J.P.); sara.valvez@ubi.pt (S.V.)

**Keywords:** composite laminates, hostile solutions, experimental tests, low velocity impact, fatigue

## Abstract

Due to the enormous benefits inherent to composite materials, they have been widely used in the most diverse fields of engineering. Therefore, it is not surprising that in many of these applications they can be exposed to hostile environments, which can affect the mechanical performance of such materials. Therefore, the main goal of this work was to study the effect of immersion into different hostile solutions on the impact strength and, subsequently, to evaluate the residual fatigue life. For this purpose, the specimens were initially immersed into solutions of hydrochloric acid (HCl), sodium hydroxide (NaOH), sulphuric acid (H_2_SO_4_), diesel, distilled water, and seawater. Subsequently, the specimens were subjected to impact loads with an energy of 12 J and, finally, subjected to fatigue loads to assess the residual fatigue life. Seawater and NaOH solution provided the lowest impact strength. This was confirmed by the lower energy restored and impact bending stiffness (IBS), a parameter that allows evaluating the damage resistance of a composite. In terms of restored energy, for example, the seawater promoted a decrease around 30.4% in relation to the value obtained with non-immersed samples, while this value was 27.6% for the alkaline solution (NaOH). In terms of IBS, the lowest values were also obtained with these solutions (437.4 and 444.9 N/mm, respectively). Finally, the lowest residual fatigue life was also observed for these two solutions, and it was noticed that there was a direct relationship between the IBS and the residual fatigue life.

## 1. Introduction

Fibre-reinforced composites are used in a wide range of engineering applications, as a consequence of their stability, high specific strength and stiffness, good static and dynamic properties, and competitive cost. Aircraft, space, automotive, sport, marine industries, and military applications are some of the fields where these materials are used [1,2,3,4]. Composites reinforced with Kevlar fibres have been widely used as an impact-resistant reinforcement in composite materials and, in this context, they are subjected to the most diverse environmental conditions with consequent effects on their mechanical properties. Several investigations about the impact of such environments on the mechanical properties of composite materials are published in the open literature. For example, regarding to flexural properties, Mahmoud and Tantawi [5] showed that the flexural strength is insensitive until 30 days after immersion into HCl, after which there is a 10% decrease. Amaro et al. [6] investigated the effects of alkaline (NaOH) and acid (HCl) solutions in fibre-reinforced polymer composites and found that flexural strength and modulus decrease with exposure time. These authors also concluded that alkaline solutions are considered more aggressive than acid ones, resulting in lower flexural properties. The effect of other acid solutions was studied by the same authors in another study [7], and it was observed that after 36 days of immersion into HCl and H_2_SO_4_ solutions, the flexural strength decreased by around 16.2% and 11.6%, respectively. The flexural modulus showed a similar trend, with values around 22.4% and 17.6%, respectively.

Several studies have been carried out on composite laminates subjected to a combination of loads and exposure to hostile environments, in which stress corrosion occurs [8,9,10,11,12]. Under these conditions, and according to Kawada and Srivastava [12], sharp cracks appear that, when propagated, facilitate the contact of the aggressive environment with the fibres. Consequently, collapse can spontaneously occur for very low load values because the mechanical strength of the fibres significantly decreases. In addition to the matrix being decisive in consolidating the structural integrity of a composite, it is also very important in adapting these materials for applications subject to hostile environments. For example, according to Banna et al. [13], the polyester resin, when exposed to harsh environments, has a lower modulus than bisphenol A epoxy vinyl ester.

It is well known that laminate composites are strong in the in-plane loading direction, but very weak in the out-of-plane loading direction. Basically, these materials have two types of damage mechanisms: interlaminar and intralaminar. Delamination, for example, is an interlaminar damage process described as the starting point and propagation of a fracture that separates two laminas and has a very significant influence on the out-of-plane and bending properties of any composite structure [14,15,16]. Matrix cracking, fibre breaking, and fibre/matrix debonding as well as fibre pull-out are some of the mechanisms of intralaminar damage that occur within the lamina [17,18,19,20] and which are closely related to low-velocity impacts [21]. In addition to being difficult to detect with visual inspections [22], they significantly affect the residual mechanical properties of these materials [23,24,25,26].

However, the literature is not abundant in studies that address impact loads and hostile environments. Mahmoud et al. [5], for example, studied the Charpy impact strength of glass/polyester composites and observed a small decrease (about 5%) after the first 60 days of immersion into HCl, but this value decreased to 10% with immersions between 60 and 90 days. However, according to Amaro et al. [6,7], the impact strength of glass/epoxy composites is highly dependent on the type of corrosive environment and exposure time. The alkaline solution proved to be more aggressive than the acid solution and, consequently, promoted a lower impact strength. However, comparing the effect of hydrochloric acid (HCl) with that of sulphuric acid (H_2_SO_4_), the first solution affected the impact performance much more. Mortas et al. [27] analysed the low-velocity impact response of Kevlar/epoxy and carbon/epoxy laminates after immersion into hydrochloric acid (HCl) and sodium hydroxide (NaOH), concluding that both corrosive environments significantly affected the impact strength, but its effect was strongly dependent on the concentration of the solutions. The authors also found a considerable effect of temperature on impact strength, regardless of the solution.

In terms of residual properties after impact, the literature focuses mainly on compressive strength because this mechanical property significantly affects the application of these materials in primary structures. On the other hand, the residual tensile strength mainly depends on the extent of fibre breakage [28,29], because delaminations have little effect on the tensile strength of laminates [25]. According to the studies developed by Reis et al. [30], increasing the impact energy decreases the residual tensile strength due to induced damage. For example, compared to the ultimate strength of non-impacted laminates, an energy of 21 J promoted a decrease in residual strength around 57.7% due to extensive damage observed in the fibres. Finally, regarding the post-impact fatigue, Chen et al. [31] found that the damage caused by the impact loading increased during compression fatigue, and three distinct stages were defined. After a small increase, damage increases at a steady rate until the third stage is reached. In this regime, it grows abruptly until the final collapse. Fatigue tests were performed by Melin et al. [32] in constant amplitude tension–compression loading on impact damaged carbon fibre/epoxy laminates, and they noted that R-value had a small influence on the fatigue life. This constitutes clear evidence that the compressive part of the load cycle is more harmful than the tensile one, which was supported by the authors’ observation. In fact, local buckling around the damage zone caused by the compression load was observed. In a similar work, Katerelos et al. [33] performed compression-compression fatigue tests on impacted specimens and a semi-analytical model based on the strain energy release rate distribution was proposed by the authors. The comparison between experimental and predicted results indicated a good model accuracy, proving to be a good tool to estimate the direction of delamination propagation, the weaker interface, and the growth rate of the delaminated area as a function of fatigue life. Butler et al. [34] proposed another theoretical model, which predicts the fatigue limit strains with an accuracy around 4% of the experimental values. From the studies carried out by Tai et al. [35], it was noticed that the damaged area increased with increasing impact energy, while the residual tensile strength significantly decreased. In terms of tension–tension fatigue behaviour, for the same stress levels, the authors observed that the fatigue life of damaged specimens is shorter than that of undamaged ones. Furthermore, the steep slope of the SN curve observed for damaged specimens indicates that the composite is sensitive to impact loading. It was also noted that low energy impacts have a significant effect on high-cycle fatigue tests, whereas this influence is not as evident in low-cycle fatigue tests. According to the authors, fibres dominate the damage mechanisms at higher stress levels, while the influence of low-energy impacts is negligible at the fibre level. However, low-energy impacts cause defects (local delaminations, matrix cracks, interface debonding, and so on) which reduce the fatigue life of impacted composites at low stress levels. In terms of stiffness variation during fatigue life, its reduction rate was higher for impacted specimens than for non-impacted specimens.

However, regarding the residual mechanical properties after impact on composite materials previously subjected to hostile solutions, the open literature is not very abundant in studies that analyse this subject. Mortas et al. [27], for example, observed that the residual bending strength of laminates previously subjected to impact loads is much lower than that observed for non-impacted specimens, but this decrease significantly increases for samples immersed into NaOH and HCl solutions and subjected, subsequently, to impacts of 10 J. The concentration (wt.%) and temperature of these solutions were variables that proved to be very important in the observed decrease. Regardless of concentration and temperature, the lowest residual bending strength occurred for Kevlar/epoxy laminates, compared with carbon/epoxy laminates, and the alkaline solution promoted lower residual bending strength compared with the acid solution.

Therefore, because impact loads introduce damage with different severities in composite materials, among which delaminations are quite dangerous because they considerably affect the mechanical properties [23,24,25,26,36,37] and are not easy to be visually detected [22,38], this study aimed to analyse the additional effect of exposure to different hostile solutions on the impact strength of Kevlar/epoxy laminates. For this purpose, immersions into hydrochloric acid (HCl), sodium hydroxide (NaOH), sulphuric acid (H_2_SO_4_), diesel, distilled water, and seawater were considered. These solutions were selected because composites are increasingly replacing traditional metallic materials in the most diverse fields of engineering. In terms of marine applications of fibre reinforced composites [39], their contact with saltwater is inevitable, which evidences the relevance of studying this fluid. In terms of diesel, its effect was analysed because fibre reinforced polymers (FRPs) are replacing steel in tanks due to the improvement obtained at level of chemical properties and weight savings compared with steel [40,41]. In relation to the other solutions, they intend to simulate the different environments that can be found in the civil engineering sector or in the chemical and food industry [7,42,43,44]. For example, while HCl is a monoprotic acid and sulfuric acid a diprotic acid, both are highly corrosive and strong mineral acids with a vast application in the chemical industry [7]. Finally, after the impact events, the fatigue life was assessed.

## 2. Materials and Methods

**Figure 1:**
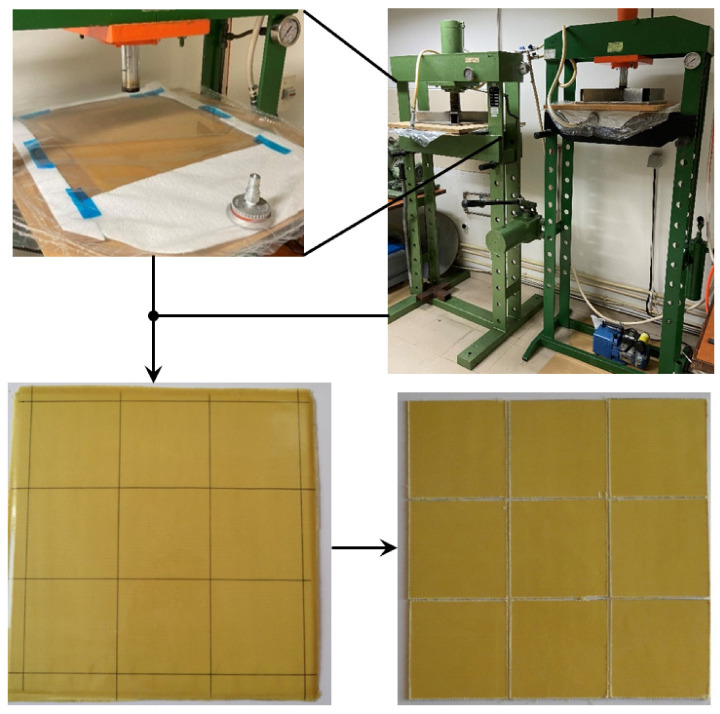
Details of the manufacturing process.

Nine ply laminates of Kevlar bi--directional woven fabrics (taffeta with 281 g/cm^2^), all in the same direction, and an Ampreg 22 epoxy resin with an Ampreg 22 hardener standard, both supplied by Gurit, were used to produce composite laminates. Plates with overall dimensions of 330 × 330 × 3.3 ± 0.1 (mm^3^) were produced by the hand lay-up process. As shown in Figure 1, this system was placed inside a vacuum bag and a load of 2.5 kN was applied for 48 h to maintain a constant fibre volume fraction and a uniform laminate thickness. During the first 10 h the bag remained attached to a vacuum pump to eliminate any air bubbles existing in the composite. The post-cure was carried out in an oven at 45 °C for 48 h.

The samples used in this study were cut from these plates to square specimens with 100 mm side (see Figure 1), which were completely submerged into different solutions and different immersion times, both summarised in Table 1. All solutions had a concentration of 10% by weight (wt.%), which corresponds to a pH of 13.0 for NaOH and 1.5 for acids. All the solutions in which the specimens were immersed were at room temperature. Finally, the samples were washed with clean water and dried at room temperature. 

It should be noted that both faces of all composites were exposed to hostile environments; however, in real conditions, only one face is exposed to these hostile environments.

**Figure 2:**
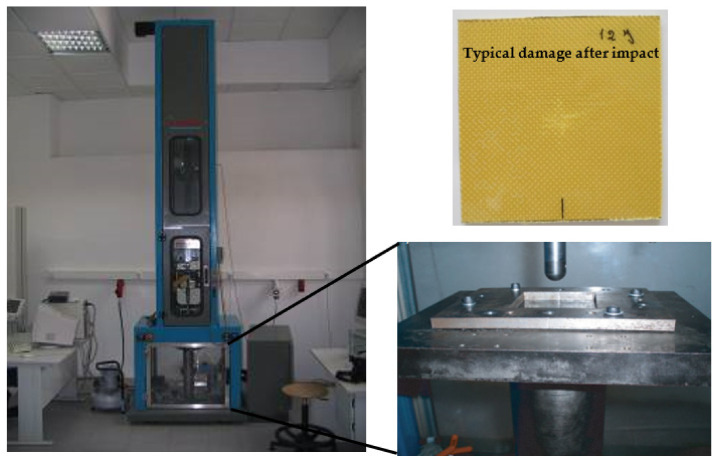
Details of the drop weight testing machine and a specimen with typical damage obtained after an impact with 12 J and immediately before immersion in hostile solutions.

Low-velocity impact tests were performed using the drop weight testing machine IMATEK-IM10 shown in Figure 2. More details of the impact machine can be found in [45]. An impactor diameter of 20 mm with a mass of 3.005 kg was used. The tests were performed on square section samples of dimensions 75 × 75 mm and the impactor stroke at the centre of the samples obtained by centrally supporting the 100 × 100 mm specimens.

Impact energies of 4, 8, 10, 12, 16, 20, 24, and 28 J were used to analyse the impact strength. This range of energies was selected to obtain damage with different severities and thus obtain an effective characterisation of the impact strength of the laminate. They were previously selected to enable the measurement of the damage area, but without promoting the perforation of the specimens [30]. From this analysis, the effect of hostile solutions on the impact strength was evaluated for the energy of 12 J. Of all the energies, 12 J was chosen because it induces much greater damage than the defects introduced during the manufacturing process [30] and, consequently, the latter were neglected in this analysis. On the other hand, according to Tai et al. [35], the severity of the different damages will be sufficient to assess their effects on long fatigue lives. Three specimens were used for each condition, and the results presented in terms of average values.

**Figure 3:**
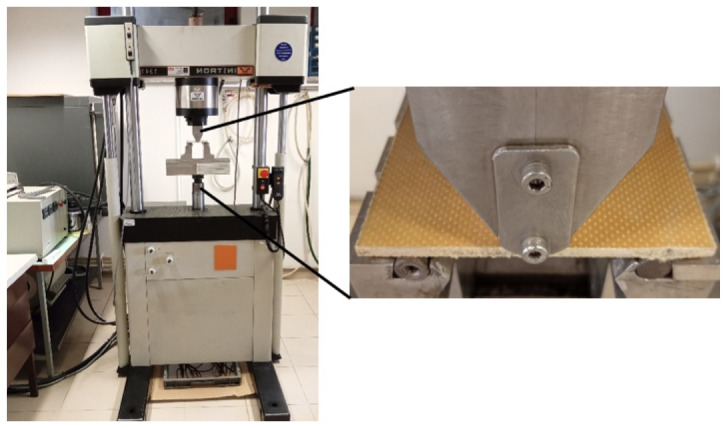
Details of the equipment used in fatigue testing.

After impact tests, the specimens were submitted to fatigue tests to assess the residual fatigue life after exposure to different hostile solutions. As shown in Figure 3, the specimens were tested with a span of 60 mm, using an Instron servo-hydraulic testing machine (model 1341), and the tests were carried out at room temperature, under constant amplitude sinusoidal waveform loading, a stress ratio of R = 0.05, and a frequency of 3 Hz. The damage criterion was established when the loss of the maximum stress reached 25% of the initial value.

## 3. Results and Discussion

**Figure 4:**
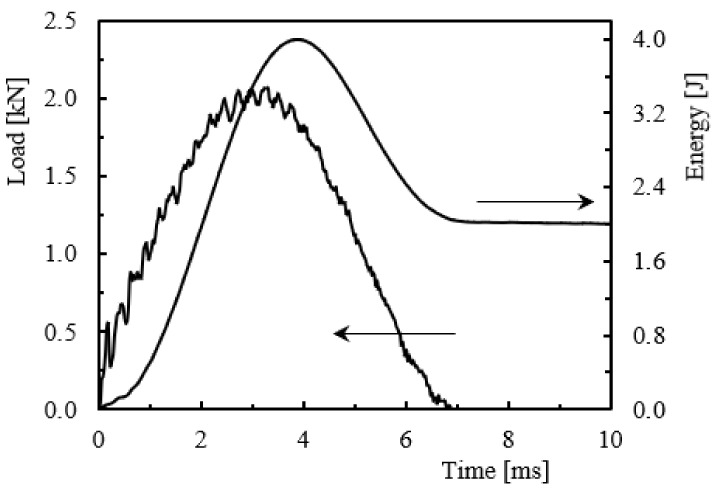
Typical load and energy versus time curves for an impact energy of 4 J.

The impact strength of Kevlar/epoxy laminates was evaluated by low-velocity impact tests performed for different impact energies. Figure 4 shows representative load-time and energy-time curves, both obtained from impact tests performed at 4 J. These diagrams represent the typical profile of all tests, which are in good agreement with those reported in the literature for similar laminates [27,30].

**Figure 5:**
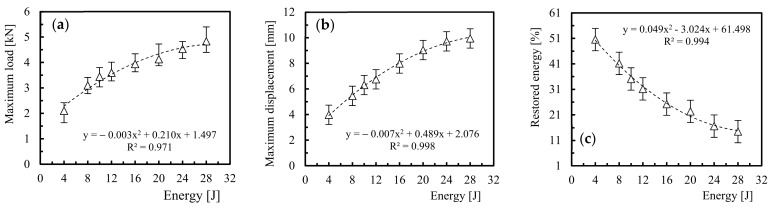
For different impact energies: (**a**) Maximum impact load, (**b**) Maximum displacement, (**c**) Restored energy.

From the load-time curves, it is possible to observe that the load increases until reaching a maximum value, P_max_, followed by a very sharp drop. These curves contain oscillations that result from the elastic wave and are created by the vibrations of the samples [30,46]. In relation to the energy-time curves, the beginning of the plateau corresponds to the loss of contact between the impactor and the specimen, which shows that the impact energy was not enough to fully penetrate the specimens. In this context, the impactor hit the sample and always rebounded. Therefore, the elastic recovery, also defined in many literature texts as restored energy, is obtained by the difference between the impact energy and the energy given by the plateau [47]. Finally, the response of laminates to different energy levels is shown in Figure 5 in terms of maximum impact load (Figure 5a), maximum displacement (Figure 5b), and restored energy (Figure 5c). Symbols represent the average values, while the bands correspond, respectively, to the maximum and minimum values obtained for each condition tested.

In terms of maximum impact load (Figure 5a), it is possible to observe that, up to 12 J, this parameter increases with increasing impact energy, but for higher impact energies this increase is more modest. Basically, the increase follows a polynomial law of degree 2, where the maximum load increases around 71% between the energies of 4 J and 12 J and only around 35% between 12 J and 28 J. This is in line with the open literature [30,48], in which it is reported that the maximum impact load increases with increasing the impact energy. According to Hosur et al. [49], the maximum impact load should increase almost linearly with increasing impact energy, but as this parameter reflects the maximum value that a composite can tolerate before the most severe damage occurs, this linearity cannot always be observed as reported, for example, in the study developed by Reis et al. [48]. Therefore, based on the study developed by Gustin et al. [50], it is possible to say that the differences observed in the maximum loads are a consequence of the different failure modes introduced in the laminate. In this context, it is possible to propose an empirical equation capable of characterising the evolution of the maximum load with the impact energy, which can be expressed by Equation (1) for the range of energies studied:

(1) $$ML=-0.003\times E^{2}+0.21\times E\;+1.497$$

where *ML* is the maximum load and *E* the impact energy value.

The influence of the impact energy on the maximum displacement is shown in Figure 5b. It is noticed that the maximum displacements increase with the impact energy and, for the range of impact energies studied, the maximum displacement increased by around 150%. Once again it is possible to propose an empirical equation capable of characterising the evolution of the maximum displacement with the impact energy, which can be expressed by Equation (2) for the range of energies studied:

(2) $$MD=-0.007\times E^{2}+0.489\times E\;+2.076$$

where *MD* is the maximum displacement and *E* the impact energy value.

Finally, Figure 5c shows that the restored energy is never equal to zero, which means that the absorbed energy is never equal to the impact energy. Therefore, the full penetration was not reached because the excess energy is used to rebound the impactor. On the other hand, it is possible to observe that higher energies promote lower restored energy (defined also as elastic recuperation) which, according to Amaro et al. [6,7], is consequence of the greater damage caused by the impact loads. For example, considering the range of energies studied, the restored energy decreased around 71.5%, which shows the severity of the damage introduced into the composite laminate. As defined for the maximum load and maximum displacement, it is possible to propose an empirical equation capable of characterising the evolution of the restored energy with the impact energy, which can be expressed by Equation (3) for the range of energies studied:

(3) $$RE=0.049\times E^{2}-3.024\times E\;+61.498$$

where *RE* is the restored energy and *E* the impact energy value.

**Figure 6:**
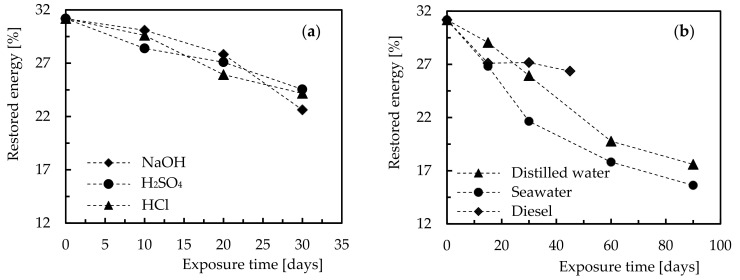
Influence of the exposure time on the restored energy for (**a**) NaOH, H_2_SO_4_, and HCl; (**b**) Distilled water, seawater, and diesel.

Given the correlation that can be established between the restored energy and the severity of the damage introduced by the impact loads [6,7], Figure 6 shows the effect of exposure time and type of solution on this parameter (restored energy). For comparison purposes, Table 2 presents the average values, and respective standard deviations, for an immersion time of 30 days into the different hostile solutions.

From Figure 6, it is possible to observe that longer exposure times promote lower values of restored energy, and there are more aggressive solutions than others. These results are aligned with those published in the open literature [6,7,27], which can be explained by the absorption, penetration, and reaction occurred between solutions and composite constituents [5]. While for the matrices the appearance of micro-cracks is a consequence of the penetration of the solution into the resin [12], in terms of fibres their interfaces with the matrix suffer degradation due to dehydration of the matrix and penetration of solutions through the micro-cracks [51,52] or voids in the matrix [12]. In fact, the degradation of the fibre-matrix interface significantly affects the load carrying capacity of the composite laminates [52], which explains the lower amount of restored energy after exposure to such hostile solutions [6,7]. This evidence is corroborated by the studies performed by Pavan et al. [53], Gargano et al. [54], and Tual et al. [55] in which the lower mechanical performance is explained by the hydrolysis, cracks due to swelling, debonding of the fibre/matrix interface, and plasticisation of the matrices. However, of all the phenomena previously reported, Tual et al. [55] proved that matrix plasticisation and degradation of the fibre/matrix interface are the most relevant, after studying the interlaminar shear strength (ILSS) of carbon/epoxy composites when immersed into water. This conclusion has been proven by other authors [56,57], including at level of impact strength [58].

Finally, from Table 2, it is possible to observe that the highest decrease in restored energy compared with the control samples (not immersed) occurred for samples immersed into seawater (30.4%), followed by samples immersed into NaOH (27.6%) and HCl (23.1%). However, when the sulphuric acid solution is compared with hydrochloric acid, the last one promoted the worst results, proving to be, in this case, a more aggressive solution. All these results are similar to those found by Amaro et al. [6,7]. 

However, according to Amaro et al. [6,7], the severity of a damage is directly related to the absorbed energy and inversely related to the impact bending stiffness (IBS). Therefore, the impact bending stiffness (IBS) is an important property to assess the damage resistance of a composite and is defined by the slope of the ascending section of the load-displacement curve. More details about this property can be found in [59]. For comparison purposes, Table 3 shows the observed decrease in impact bending stiffness after 30 days of immersion into different solutions compared to the value obtained for the control samples.

As reported above, lower IBS values are synonymous of larger damages, which, according to Table 3, occurred in laminates that were immersed into seawater and into NaOH and HCl solutions. On the other hand, the highest IBS value is obtained for non-immersed samples (control samples), although samples immersed into distilled water and diesel have very close values, which means that the severity of the induced damage was not very large compared with the introduced by the impact loads on the control specimens.

**Figure 7:**
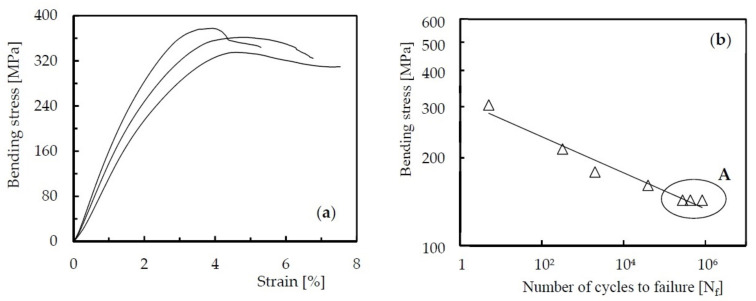
Flexural stress properties (**a**) and SN curve (**b**) for Kevlar/epoxy laminates after an impact of 12 J.

Finally, to observe the effect of the severity of such defects on the residual fatigue life, Figure 7 shows the curves that allow the static characterisation of Kevlar laminates after impact (Figure 7a), as well as the fatigue characterisation using the typical SN curves (Figure 7b). From the static curves shown in Figure 7a, it is possible to observe a linear regime up to about 160 MPa, a value from which there is a non-linear regime that contains the maximum bending stress. In this case, the maximum bending stress of the laminates after impact is around 358 MPa, with a standard deviation of 22 MPa. On the other hand, Figure 7b) shows the SN curve that characterises the fatigue behaviour of laminates after impact. As expected, the increase in bending stress promotes a decrease in fatigue life and the behaviour observed is in good agreement with the fatigue studies reported in the literature for Kevlar laminates [60,61]. According to these studies, the fatigue strength significantly depends on the defined failure criterion, because the total rupture of the specimens was not observed. In this case, it is necessary to adopt a criterion of failure (in the present study, this was assumed when the loss of maximum stress reached 25% of the initial value). It has been shown that fatigue life dependents on the amplitude of the applied load as well as its maximum value. Finally, because the aramid fibres fail by a series of small fibril failures, they promote long fatigue lives and do not cause the abrupt/brittle collapse of the composite. On the contrary, they promote significant delaminations between layers.

Literature reports that defects severely affect the mechanical properties of any composite, of which voids have a particular relevance because they are inherent to manufacturing processes. Such defects have special importance on the mechanical properties dominated by the matrix material [62], because they decrease the interlaminar shear strength, reduce the cross-sectional area or, if they are sufficiently large, promote the propagation from an individual void [63,64,65]. However, as shown in a previous study [30], the damage introduced by the 12 J impact was much larger/severe than the defects introduced along the manufacturing process and, in this case, it will be the dominant damage that will propagate when cyclic loads are applied. On the other hand, according to Tai et al. [36], the fatigue life of impacted specimens is shorter than that of undamaged specimens and the damage effect is more expressive for longer fatigue lives than for shorter lives (where higher fatigue loads are used). Therefore, to evaluate the real and expressive effect of the hostile solutions on the residual fatigue life, the lowest maximum bending stress (143 MPa) was considered in order to expect longer fatigue lives. In fact, fibres dominate the damage mechanisms at higher stress levels, but for the stress level considered only the damage propagation induced by the impact load is expected.

**Figure 8:**
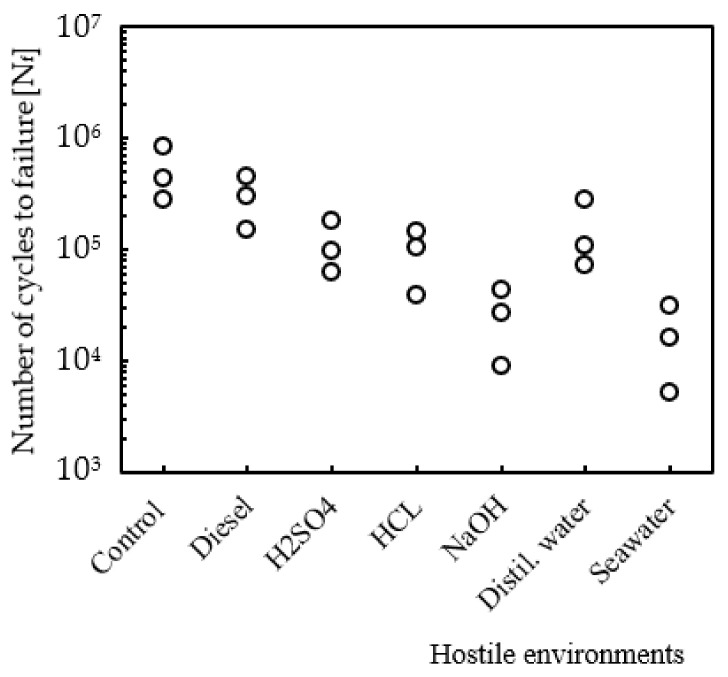
Residual fatigue life for laminates immersed into different hostile solutions.

Based on these considerations, Figure 7 essentially represents the effect of impact damage on static strength and fatigue life of non-immersed laminates (control specimens), while Figure 8 shows the residual fatigue life for laminates immersed into different hostile solutions. The comparison was established for all conditions tested with the same bending stress of 143 MPa (A), for the same exposure time of 30 days, and the symbols (circles) shown in the figure represent the fatigue life obtained for each specimen. For control samples after impact, the average residual fatigue life is about 520,704 cycles, but, according to Figure 8, an exposure to seawater during 30 days promotes a decrease of around 96.6%, while for solutions of NaOH and HCl the reduction was only 95% and 81.6%, respectively. For the other solutions, the decrease was not so abrupt, assuming, for example, values around 78%, 71.1% and 42.6%, respectively, for H_2_SO_4_, distilled water and diesel.

According to Amaro et al. [6], the degradation of the matrix/fibre interface as well as the matrix stiffness explain the decrease observed in the fatigue life. In fact, several studies report that the matrix stiffness decreases when exposed to these solutions, and the decrease increases with the exposure time and concentration of the solutions [6,7,27]. These results are in good agreement with those reported above, from which it is possible to conclude that lower values of impact bending stiffness (IBS) are related to more severe damage and, consequently, to shorter residual fatigue lives.

**Figure 9:**
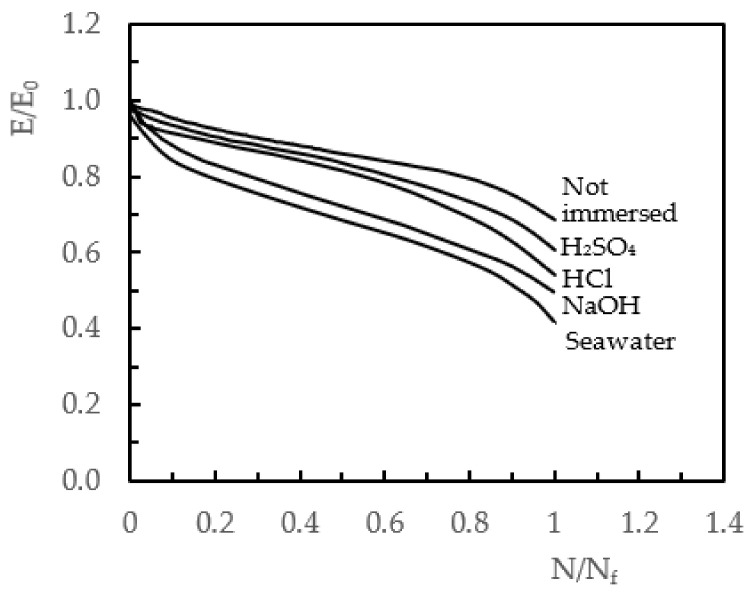
E/E_0_ against the normalised number of cycles (N/N_f_) for different hostile solutions.

The loss of stiffness during fatigue tests was investigated as a damage criterion by several authors because, according to them, this parameter is directly related to the damage and its evolution/propagation [66,67,68,69,70]. Therefore, to understand the effect of the hostile solutions on the fatigue life, Figure 9 plots E/E_0_ versus N/N_f_, where E is the instantaneous stiffness modulus, E_0_ is the initial modulus at beginning of the test, N is the number of cycles at any given instant of the tests, and N_f_ is the number of cycles to failure.

For comparison, only solutions of NaOH, H_2_SO_4_, HCl, seawater, and non-immersed specimens (control samples) were considered and, regardless of the hostile environment, the observed curves presented three distinct stages. Initially, there was a very significant loss of stiffness in the first 5% to 10% of fatigue life, followed by a second stage that was characterised by a slow decrease in stiffness modulus until close to final failure. Finally, during the last 5% to 10% of the fatigue life, the third stage was characterised by the sudden drop in the modulus of stiffness. According with the open literature, this behaviour is explained essentially by the damages and their propagations [68,69,70]. However, as reported above, fatigue tests were performed on specimens with damage of different severities, as evidenced in the IBS analysis. In this context, more severe defects propagate faster in the laminate and, as expected, shorter fatigue life occurs.

For example, considering the non-immersed samples, the slope of the curve corresponding to the second stage was much smaller than that observed for samples immersed in seawater. This is clear evidence that the damage introduced by impact loads on laminates immersed in seawater is much more significant than that obtained on laminates impacted without having been exposed to any solution. Consequently, the damage spread faster during the fatigue loading and shorter lives were observed. Finally, it is also possible to note that there is a direct relationship between the residual fatigue life and the impact bending stiffness (IBS), i.e., lower IBS values correspond to shorter fatigue lives.

## 4. Conclusions

This work studied the low-velocity impact response of a Kevlar fibre/epoxy laminate after immersion into diesel, H_2_SO_4_, HCl, NaOH, distilled water, and seawater. It was possible to conclude that the impact properties are significantly affected by hostile environments, with alkaline solution and seawater being the most aggressive. Moreover, the exposure time proves to be determinant, where it was noticed that the impact properties decreased with the increase of the exposure time. Impact flexural stiffness (IBS) proved to be an important property in evaluating the effect of hostile solutions on the impact performance of composite laminates. Finally, the severity of the damage promoted the shortest residual fatigue life, and it was possible to observe a direct relationship between the IBS and the residual fatigue life.

## Figures and Tables

**Table 1:** Different solutions and immersion times used in this study.

Solutions	Immersion Time (days)
Diesel	15, 30, and 45
Sulphuric acid (H_2_SO_4_)	10, 20, and 30
Hydrochloric acid (HCl)	10, 20, and 30
Sodium hydroxide (NaOH)	10, 20, and 30
Distilled water	15, 30, 60, and 90
Sea water at room temperature	15, 30, 60, and 90

**Table 2:** Restored energy for 30 days of immersion and comparision with the value obtained for control samples.

Solutions	Restored Energy [%]	Decease Compared to Control Samples [%]
Control samples (not immersed)	31.2 (4.8)	-
Diesel	27.2 (2.6)	12.8
Sulphuric acid (H_2_SO_4_)	24.7 (3.0)	20.8
Hydrochloric acid (HCl)	24.0 (2.8)	23.1
Sodium hydroxide (NaOH)	22.6 (3.3)	27.6
Distilled water	25.9 (2.4)	17.0
Seawater at room temperature	21.7 (3.1)	30.4

Average value (Standard deviation).

**Table 3:** Impact bending stiffness (IBS) and respective comparision with the value obtained for control samples.

Solutions	IBS [N/mm]	Decease Compared to Control Samples [%]
Control samples (not immersed)	521.7 (18.3)	-
Diesel	511.2 (20.6)	2.0
Sulphuric acid (H_2_SO_4_)	490.2 (12.4)	6.0
Hydrochloric acid (HCl)	469.5 (10.9)	10.0
Sodium hydroxide (NaOH)	444.9 (15.1)	14.7
Distilled water	501.2 (19.2)	3.9
Sea water at room temperature	437.4 (18.9)	16.2

Average value (standard deviation).

## Data Availability

The data presented in this study are available within this article.

## Sample Availability

Samples of the compounds are available from the authors.
